# Biological Systems from an Engineer's Point of View

**DOI:** 10.1371/journal.pbio.1000021

**Published:** 2009-01-20

**Authors:** Gregory T Reeves, Scott E Fraser

## Abstract

The robust design of complex systems, such as cruise control, requires a careful balance of several objectives. As biological systems are no different, an engineering approach to these systems proves useful.

Mathematical modeling of the processes that pattern embryonic development (often called biological pattern formation) has a long and rich history [1,2]. These models proposed sets of hypothetical interactions, which, upon analysis, were shown to be capable of generating patterns reminiscent of those seen in the biological world, such as stripes, spots, or graded properties. Pattern formation models typically demonstrated the sufficiency of given classes of mechanisms to create patterns that mimicked a particular biological pattern or interaction. In the best cases, the models were able to make testable predictions [3], permitting them to be experimentally challenged, to be revised, and to stimulate yet more experimental tests (see review in [4]). In many other cases, however, the impact of the modeling efforts was mitigated by limitations in computer power and biochemical data. In addition, perhaps the most limiting factor was the mindset of many modelers, using Occam's razor arguments to make the proposed models as simple as possible, which often generated intriguing patterns, but those patterns lacked the robustness exhibited by the biological system. In hindsight, one could argue that a greater attention to engineering principles would have focused attention on these shortcomings, including potential failure modes, and would have led to more complex, but more robust, models. Thus, despite a few successful cases in which modeling and experimentation worked in concert, modeling fell out of vogue as a means to motivate decisive test experiments.

The recent explosion of molecular genetic, genomic, and proteomic data—as well as of quantitative imaging studies of biological tissues—has changed matters dramatically, replacing a previous dearth of molecular details with a wealth of data that are difficult to fully comprehend. This flood of new data has been accompanied by a new influx of physical scientists into biology, including engineers, physicists, and applied mathematicians [5–7]. These individuals bring with them the mindset, methodologies, and mathematical toolboxes common to their own fields, which are proving to be appropriate for analysis of biological systems. However, due to inherent complexity, biological systems seem to be like nothing previously encountered in the physical sciences. Thus, biological systems offer cutting edge problems for most scientific and engineering-related disciplines. It is therefore no wonder that there might seem to be a “bandwagon” of new biology-related research programs in departments that have traditionally focused on nonliving systems.

Modeling biological interactions as dynamical systems (i.e., systems of variables changing in time) allows investigation of systems-level topics such as the robustness of patterning mechanisms, the role of feedback, and the self-regulation of size. The use of tools from engineering and applied mathematics, such as sensitivity analysis and control theory, is becoming more commonplace in biology. In addition to giving biologists some new terminology for describing their systems, such analyses are extremely useful in pointing to missing data and in testing the validity of a proposed mechanism. A paper in this issue of *PLoS Biology* clearly and honestly applies analytical tools to the authors' research and obtains insights that would have been difficult if not impossible by other means [8].

## Dynamical Systems

Many dynamical systems can be sufficiently described by a set of ordinary differential equations (ODEs), which model how the variables change in time (but not space). A variable, such as enzyme concentration, could be affected by three factors: rates of production, degradation/reaction, and influx/efflux. Adding these influences together will tell us how that variable evolves.

Consider a simple example of an enzyme with intracellular concentration, *c*, and which is produced at a rate *r*_p_(*c*) and degraded or diminished at a rate *r*_d_(*c*). Assuming the concentration is uniform within the cell (a poor, yet common assumption), we can write an ODE describing how the enzyme concentration will evolve:

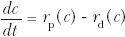



The first step in analysis is to determine the steady-state solution (the enzyme concentration that allows for the balance between the rates of production and degradation; see example in Figure 1). This is achieved by setting *dc*/*dt* = 0 (implying that concentration no longer changes in time) and solving the remaining algebraic equation.

**Figure 1:**
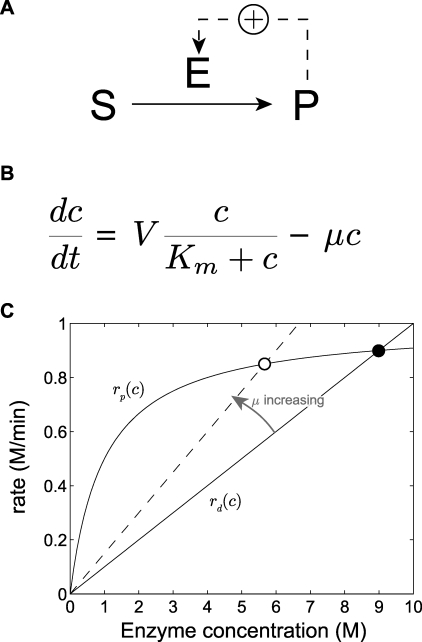
Example of a Dynamical System (A) Reaction schematic of an enzyme, E, catalyzing the conversion of substrate, S, to product, P. The final product acts as a catabolite to promote the expression of enzyme. (B) ODE describing the change in time of enzyme concentration, *c*. The first term is the production term, *r*_p_(*c*), and the second term is degradation, *r*_d_(*c*). (C) Graphical representation of *r*_p_(*c*) and *r*_d_(*c*). At steady state, these two processes will balance, and the concentration of enzyme will become constant in time, with a value corresponding to the intersection of these two curves. Increasing the degradation constant μ (depicted by shift from solid line to dashed line) changes the steady state value of *c* (from closed circle to open circle). The sensitivity of this steady state value of *c* to such changes in parameters can be quantified by the sensitivity coefficient.

To make the example concrete, consider the case in which the enzyme concentration promotes its own production (Figure 1A) [9]:

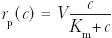
 where *V* is the maximal production rate and *K*_m_ is analogous to a Michaelis-Menten constant, and assume enzyme concentration is diminished in a first-order process (see Figure 1B). This removal rate is usually due to a combination of enzyme turnover and a dilution effect due to cell growth. In this case, we can solve the steady state equation, finding *c* = *V*/μ + *K*_m_, where μ is the decay rate constant. This can be seen graphically in Figure 1C, where the steady-state value of *c* lies at the intersection between *r*_p_(*c*) and *r*_d_(*c*). After calculating the steady state, a sensitivity analysis is often performed to determine the robustness of the solution to variations in parameter values.

## Sensitivity Analysis

In recent years, much research has focused on the robustness of biological systems [10,11]. Indeed, it is clear that biological systems must maintain several variables within a narrow limit in the face of a wide variety of external pressures.

In applied sciences, “sensitivity” (the opposite of robustness) is measured by a quantity called the *sensitivity coefficient*. This quantity measures the amount by which an output of the system (e.g., enzyme concentration) changes with respect to variation in an input quantity (e.g., degradation rate). Changes are given in fractional terms, or change relative to the nominal state. For example, changes in the decay rate constant μ by a small amount are characterized by the fractional change Δμ/μ. Similarly, the changes in enzyme concentration *c* are characterized by Δ*c*/*c*, and the ratio of these is the sensitivity coefficient. This quantity is usually measured in the case of infinitesimally small changes in input, which converts the Δ's into a derivative:

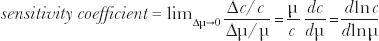
 where the last equality is a simple definition from calculus and demonstrates that the sensitivity coefficient is equivalent to the slope of a log-log plot of output versus input.

But what does this number mean? How do you know whether a value of the sensitivity coefficient is “good?” In the absence of further knowledge, we would like the absolute value of the sensitivity coefficient to be less than 1. This implies that any fractional change in the input will correspond to a smaller fractional change in the output. Conversely, if the absolute value is greater than 1, small fluctuations in the input will be amplified in the output.

To illustrate an application of this analysis, we return to our example in Figure 1. If the decay constant, μ, is increased, the solid line shown in Figure 1C shifts to the dotted line. Correspondingly, the steady state value of *c* decreases to the open circle. Using parameter values of *V* = 1 M/s, *K*_m_ = 1 M, and μ = 0.1 s^–1^, it is easy to calculate *d*ln*c*/*d*lnμ = –1.11. As already noted, this is undesirably high (magnitude greater than 1); changes in μ near μ = 0.1 M/s will be amplified by 11%.

To make matters worse, it is easy to show in this illustration that no feasible values of the three parameters can give a sensitivity coefficient of magnitude less than 1. In general, it may be difficult to find parameter values that ensure robust outputs. In engineered systems, this is often solved by feedback regulation.

## Thinking Like an Engineer

The engineering and applied mathematics subfield of “control theory” refers to the use of feedback loops to ensure that system outputs, such as product purity, are maintained at set values (see general control loop in Figure 2A). Cruise control (now standard on most cars) is an everyday example of a feedback control system. When at the desired speed, the driver implements the set point by pushing the “set” button. Thereafter, the controller calculates the difference between the current speed and the set point, and opens or closes the throttle accordingly (Figure 2B). In reality, the controller uses not only the current difference (proportional control), but also the time history of the difference (integral control) and how fast that difference is changing (derivative control) to decide how strongly to respond. As each of the control strategies has inherent advantages and disadvantages, rarely do engineers implement only one control strategy at a time; most often controllers are of the PI (combining proportional and integral) or PID (combining all three) type.

**Figure 2:**
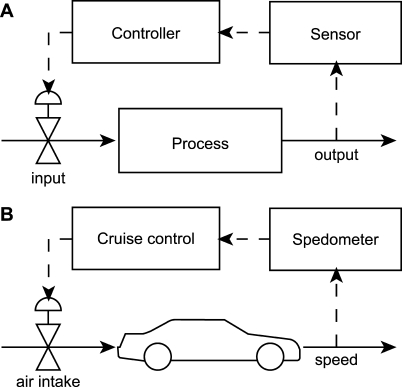
Examples of Control Loops (A) Schematic of a simple control loop. The process output is monitored by a sensor, and the value of this output signal is passed to a device called a controller. The controller calculates the difference between the output signal and the set point (the desired value of the output), and responds accordingly, often by physically manipulating an input parameter, such as a control valve. (B) Schematic of cruise control. The car is the process, and the car's speed is the output. A speedometer sensor within the car tells the cruise control the car's speed. The actuator on the cruise control then responds by opening or closing the throttle, allowing air intake into the engine.

Use of feedback control always comes with a cost, or design trade-off: an increase in robustness/performance in one area must be accompanied by a decrease somewhere else [12]. Therefore, when designing a system, an engineer (reluctantly) adds complexity only when convinced that the simpler system cannot achieve the desired ends. Because of design trade-offs, the best an engineer can do to improve a system through control is to transfer the sensitivity from a critical variable to one not-so-critical. As an example, the cruise control in your car ideally will respond to differences in speed quickly (but not too quickly). On the other hand, a fast-responding controller is sensitive to overshooting the desired speed (and in the worst case, to unstable oscillations around the desired speed). This can be suppressed, but only at the expense at being more sensitive to noise. In a very real sense, plugging a hole with your finger simply results in a leak sprung somewhere else.

At this point, it is instructive to stop and consider the analogy between biological tissues and engineered systems (such as a car). Many biologists have remarked on the apparent design of biological systems, arguing that this is a false analogy. However, evolutionary theory would predict apparent design and purpose in biological systems. Therefore, regardless of the origin of this apparent design, the analogy is, at the very least, pragmatic [13,14]. Keeping this in mind, we can approach a biological system from an engineer's perspective. Engineered systems were designed with a particular purpose in mind, so it would be helpful to ask, “What is/are the purpose(s) of this biological system?” Lander has called these purposes “performance objectives,” and determining what they are for a particular biological system is especially important in light of design trade-offs, and furthermore will provide clues to a system's molecular behavior [14].

To demonstrate, let us return our simple example in Figure 1. We had originally assumed the removal rate of the enzyme was first-order, partially due to a dilution effect: enzyme concentration decreases as the cell grows in volume. However, if the enzyme is responsible for a growth-limiting reaction, the growth rate itself is proportional to the enzyme concentration, and thus the degradation rate becomes quadratic: *r*_d_(*c*) = μ*c* ^2^. In a sense, this is a type of negative feedback in which the enzyme is responsible for its own dilution. Under these conditions, the sensitivity coefficients for all of the parameters will always have a magnitude less than 1! However, as a tradeoff, the system will generally take a longer time to reach steady state. So which performance objective is more important to the cell: robustness with respect to parameter variation, or rapid approach to steady state?

In other words, when optimizing a large system with several performance objectives, any one objective, taken in isolation, is simple to optimize. However, optimization of the whole system—that is, balancing multiple performance objectives—can be extremely difficult to achieve, especially because performance objectives are often at odds with each other. Regarding robustness, in most cases (outside of simple examples with one variable and three parameters), it is not feasible to design a system in which all sensitivity coefficients have a magnitude of less than one; more information is needed to ascertain the tolerable values of the sensitivity coefficients. What levels of fluctuations in the inputs are we expected to face? What kind of error in the output variable(s) are we prepared to tolerate? It would be a poor performance objective to be robust to variables that are either well-controlled or have narrow windows of natural variation, or to minimize the sensitivity of an output that has no need of tight control.

Furthermore, robustness to some variables may have disastrous consequences. Consider the example of cruise control, discussed earlier. If we focus on the controller (rather than the car) as the system, then the input is the speed of the car, and the output is the signal that opens or closes the throttle. If the controller itself were resistant to changes in speed of the car, the control system would be useless! In other words, determining performance objectives is a first major step in understanding the function of a biological system.

## A Prime Example

In this issue of *PLoS Biology*, Lander and colleagues illustrate the utility of taking an engineer's perspective in the context of the olfactory neuron cell lineage in the mammalian olfactory epithelium (OE) [8]. They describe this unbranched cell lineage as consisting of stem cells, an intermediate stage of transit-amplifying precursors, and terminally differentiated olfactory receptor neurons (ORNs). They investigate the mechanisms by which this tissue self-regulates its size, during both development and regeneration following injury. According to the reigning hypothesis, the ORNs secrete the inhibitors GDF11 and activin, causing longer cell cycles of stem and precursor cells. Thus, as the tissue grows, inhibitor concentration increases, slowly stalling and eventually ceasing cell division. Any loss of tissue results in a decrease in inhibitor concentration, leading to a proliferative phase for the stem and precursor cells to replace the lost tissue.

The authors begin with a general analysis of unbranched cell lineages, using a set of ODEs to model the population of each cell stage. In the case with no feedback regulation in the olfactory neuron cell lineage, they show that the system can only realize a steady state number of ORNs under very special conditions (i.e., parameter values). A sensitivity analysis shows that even small deviations from these conditions have drastic consequences on the steady state.

In further analysis, Lander et al. focus on several experimentally observed performance objectives—including rapid regeneration after injury, low progenitor load (stem plus precursor cells make up less than 10% of the OE), and robustness of the steady state—and ask whether feedback loops could be designed to simultaneously meet these objectives. Briefly, they find that GDF11 must act not only to slow the cell cycle of precursor cells, but also to increase the likelihood that a dividing precursor cell may produce ORNs. In a similar manner, activin must also act on the stem cell population.

Next, the authors investigated the ability of the OE to sense varying concentrations of GDF11 and activin. In this case, the performance objective is *sensitivity* of inhibitor concentration to tissue size (recall the example of the controller on a car being sensitive to the car's speed). They demonstrate that two factors must be finely tuned to achieve this objective: (1) the decay length of the inhibitor (i.e., how far it can travel before being degraded) compared to average tissue height, and (2) the ratio of the decay length inside the OE to that outside (in the basal stroma).

Remarkably, the work done by Lander et al. [8] suggests that biological systems achieve control in much the same way as do engineered systems. In the OE, GDF11 and activin act as readouts of the size of the tissue (i.e., sensors), and the “controller” can be thought of as the cellular machinery that transduces and actuates the inhibitory signal. The analogy may not merely be superficial, as the authors speculate that each of the types of control strategies (proportional, integral, and derivative) is used in some fashion in the OE.

## What Does This All Mean?

Many biologists have now begun to advocate mathematical modeling, noting that “a cartoon model” is no longer sufficient [5]. On the other hand, some readers would be skeptical of results that so heavily focus on the mathematics. This is not an unfounded skepticism, as evinced by numerous mathematical models in biology that have failed to accurately describe experimental systems. Indubitably, even the best mathematical models fail to incorporate full mechanistic detail of biological systems. However, when derived from well-established physics and chemistry, these models can reveal general system behavior that is not easily supported by intuitive reasoning.

This is the case with the study by Lander et al. The authors begin with very general arguments from physics and mathematics, which correctly describe the overall behavior of a cell lineage without feedback, and have no need to model further detail. Indeed, a more detailed model would necessarily behave in a qualitatively similar fashion, but would muddy the water on the conclusions. Noting that the simple model fails to display robustness (a critical performance objective), they add complexity step-wise until this performance objective is met. Their model is sufficient to show that feedback is vital to the stability and robustness of any such lineage. While few would be surprised at this, the authors also show what type of feedback is necessary to achieve the stated performance objectives of rapid regeneration.

Some have argued that a mathematical model is of no use unless it is able to make further predictions about the system. To do this, it is preferable to include mechanistic detail in the model [5,6]. Should we say then that it is useless to analyze biological systems without such detail? Clearly, the answer is no, as the model presented by Lander et al. has revealed new insights. While the authors make (and experimentally investigate) some model predictions, they argue that their main goal was to use modeling as an explanatory tool and to approach this system from an engineer's perspective. It seems they have met their performance objectives.

## Abbreviations

ODE: ordinary differential equation
OE: olfactory epithelium
ORN: olfactory receptor neuron
